# Climate Action for Health: An Urgent Call from the Global Cardiovascular Community

**DOI:** 10.5334/gh.1051

**Published:** 2021-05-03

**Authors:** Fausto J. Pinto, Kelcey Armstrong-Walenczak, Karen Sliwa

**Affiliations:** 1 Universidade de Lisboa, PT; 2 World Heart Federation, CH; 3 University of Cape Town, ZA

**Keywords:** air pollution, cardiovascular disease, cvd, environmental health impacts, climate

## Abstract

The current and immediate past Presidents of the World Heart Federation are pleased to publish this invited editorial to demonstrate the organization’s strong, ongoing commitment to addressing the impacts of air pollution on cardiovascular health and outline its strategy for action.

Air pollution and its impact on human health has emerged as a momentous global health concern over the last several years. While the effects of air pollution on the respiratory system are widely recognized and understood, not enough attention is drawn to the fact that 50% of the estimated 6.7 million deaths attributable to air pollution in 2019 were due to cardiovascular diseases [1]. An estimated 12% of all deaths in 2019^1^ were attributable to outdoor and household^2^ air pollution, and nine out of ten people worldwide breathed polluted air. Air pollution increases the risk of heart disease [2,3,4] and stroke [5], as well as lung cancer, COPD, respiratory infections, and diabetes [6], which are known to raise a person’s risk of experiencing some of the more severe consequences of COVID-19. With these impacts, air pollution is a major contributor to the global burden of disease: it was the fourth highest ranking risk factor for mortality in 2019, with more attributable deaths than high LDL cholesterol, high body-mass index, physical inactivity, or alcohol use.

That is why the world’s leading voices in cardiovascular health – represented by the World Heart Federation (WHF), American College of Cardiology (ACC), American Heart Association (AHA), and European Society of Cardiology (ESC) – took the initiative this year to release the first joint statement of its kind urging the medical community and health authorities to take urgent action to mitigate the impact of air pollution on health.

Although the attention of the world and the global health community remains fixed on the COVID-19 pandemic, other risk factors continue to have significant impact on our health and could further contribute to the virus’ negative effects. Air pollution is one crucial example. Established evidence from other respiratory viruses and emerging evidence for COVID-19 indicate that air pollution alters respiratory defense mechanisms, leading to a more severe infection. Air pollution also contributes to co-morbidities – that is, multiple underlying health-related conditions present simultaneously – that are known to worsen outcomes amongst those infected with COVID-19.

This dangerous ‘triple threat’ of air pollution, COVID-19, and cardiovascular disease must be taken seriously. If we want to reduce global deaths from cardiovascular and other non-communicable diseases, we cannot wait for the pandemic to pass to take meaningful action on air pollution. However, addressing air pollution effectively will require a multi-system and multi-sectoral response. There are no shortcuts to achieve the ‘building back better’ refrain we so often hear these days, but there are a number of evidence-based measures that have proven to benefit health at the population level, such as improvements in active transit, investment in wind and solar energy, and stricter emissions controls on building and industrial pollutants at national and city levels across the EU, from Granada to Ljubljana [7]. Recent initiatives of the U.S.’s new Biden Administration, including the motions to re-engage with the Paris Climate Agreement and ask Congress to reduce fossil fuel subsidies, and by private industry, such as GM’s commitment to sell only zero-emission vehicles by the year 2035 [8], do much to demonstrate the political will necessary to create a virtuous cycle of cleaner air and healthier populations.

The health sector as a whole, which bears the healthcare costs from the impacts of air pollution, can also provide much-needed support for ministries of environment, energy, and transportation, which are traditionally responsible for mitigation efforts. Yet while structural actions to mitigate pollution emissions are ultimately necessary to reduce harmful exposures, health care providers can play several important roles while the mitigation measures are being developed and implemented.

First, clinicians can advocate for air pollution mitigation as a health measure, and we invite interested readers to visit the website of the World Heart Federation to learn more about how they can get involved.

Second, clinicians can provide patients with personal measures to reduce exposures and associated risk at the individual level. Health care providers can integrate air pollution into disease management approaches.

And finally, the public health sector can evaluate policies to reduce air pollution and quantify both the health benefits and healthcare cost savings.

To develop a more comprehensive response to this growing global challenge, the World Heart Federation (WHF) has convened a high-level Air Pollution Expert Group (APEG) to foster and guide its work in this area. The complexity and scale of this issue create an unfortunate lack of understanding among those with the power to make change for good, including doctors and policymakers, which in turn results in a subsequent lack of concerted action. WHF is ideally poised to address the low levels of acknowledgment and acceptance of the impacts of air pollution on circulatory health among cardiology societies, heart-health foundations, and institutes of medical education and their members working on the front lines of healthcare and health policymaking. By consolidating existing evidence to make a strong case for action, educating key target audiences across sectors and borders, and advocating for policies that mitigate and limit the negative impacts of air pollution on heart health, together the ten distinguished members of the APEG will drive progress towards the overall objective of reducing the negative health outcomes caused by the effects of air pollution on the cardiovascular system, as detailed in the group’s newly published strategy. An official policy brief will follow.

Despite the massive disruption of the COVID-19 pandemic, there are reasons for optimism in the movement towards cleaner air. Broad societal lockdowns have shown us a glimpse of what a future with strong air pollution measures could yield: many cities, notably New Delhi, experienced for the first time in decades a fresh breath of clean air that can serve as an ongoing goal for cross-sectoral cooperation. Greater recognition of underlying vulnerabilities and inequities that have driven disparities in COVID-19 severity can help target the contributors to poor health, and support from across disparate sectors can raise the profile of air pollution on the global political agenda. By working together to demand and enact strong policies to counter air pollution, we can envision blue skies ahead for heart health around the world.

## Footnotes

While other estimates exist and each have their uncertainties, all estimates of attributable disease burden are large and high ranking in comparison to traditional and more widely recognized CVD risk factors.

Household air pollution is mainly a concern in low income countries where polluting fuels (coal, wood, agricultural residue, animal dung) are used for cooking and heating. https://pubmed.ncbi.nlm.nih.gov/33069327/.
